# Benzo[*a*]pyrene and *Caenorhabditis elegans*: defining the genotoxic potential in an organism lacking the classical CYP1A1 pathway

**DOI:** 10.1007/s00204-020-02968-z

**Published:** 2021-01-09

**Authors:** Mustafa Abbass, Yuzhi Chen, Volker M. Arlt, Stephen R. Stürzenbaum

**Affiliations:** 1 Department of Analytical, Environmental and Forensic Sciences, School of Population Health and Environmental Sciences, Faculty of Life Sciences and Medicine, King’s College London, London, UK; 2 Present Address: Toxicology Department, GAB Consulting GmbH, 69126 Heidelberg, Germany

**Keywords:** *Caenorhabditis elegans*, Benzo[*a*]pyrene, DNA adducts, Xenobiotics

## Abstract

Benzo[*a*]pyrene (BaP) is bioactivated in most organisms by the cytochrome P450 (CYP) enzymes, mainly CYP1A1, ultimately resulting in the reactive metabolite BaP-7,8-dihydrodiol-9,10-epoxide (BPDE) capable of covalently binding to DNA and forming adducts. This step has been defined as the key process in cancer initiation in humans. However, limited knowledge is available about the consequences of BaP exposure in organisms lacking this classical CYP1A1 pathway, one example is the model nematode *Caenorhabditis elegans.* The aim of this study was to define the genotoxic potential of BaP in *C. elegans* and to advance our understanding of xenobiotic processing in the absence of the CYP1A1 pathway. Exposure to high concentrations of BaP (0–40 µM) significantly affected life cycle endpoints of *C. elegans*, which were manifested by a reduced reproductive output and shortened life span. An optimised comet assay revealed that DNA damage increased in a dose-dependent manner; however, no bulky DNA adducts (dG-*N*^*2*^-BPDE) were observed by ^32^P-postlabelling. Global transcriptomic analysis by RNA-Seq identified responsive transcript families, most prominently members of the *cyp-35* and UDP-glucuronosyltransferases (UGTs) enzyme families, both of which are linked to xenobiotic metabolism. Strains harbouring mutations in the *cyp-35A2* and *cyp-35A3* genes were notably less prone to BaP-mediated toxicity, and BaP led to longevity in *cyp-35A5* mutants. In summary, BaP induces transcriptional, genotoxic and phenotypic responses in *C. elegans*, despite the absence of the classical CYP1A1 bioactivation pathway. This provides first evidence that parallel pathways are implicated in BaP metabolism in *C. elegans* and this seems to be mediated via the *cyp-35* pathway.

**Supplementary Information:**

The online version contains supplementary material available at 10.1007/s00204-020-02968-z.

## Introduction

Polycyclic aromatic hydrocarbons (PAHs) are products of the incomplete combustion of organic matter which are present in polluted air, diesel engine exhaust and tobacco smoke (Phillips 1999). Benzo[*a*]pyrene (BaP) is classified as Group 1 human carcinogen by IARC, because it can induce DNA damage and mutations in growth-controlling genes such as tumour suppressors or oncogenes leading to tumour development (IARC 2010). BaP has been extensively studied and is often used as a model PAH to define the underlying mechanisms linked to PAH carcinogenesis (Kasala et al. 2015). As a pro-carcinogen, BaP requires bioactivation by members of the cytochrome P450 (CYP) superfamily, which entail many haem-containing mono-oxygenases. In humans and rodents, BaP is first oxidised predominantly by CYP1A1 to BaP-7,8-epoxide, which is converted by microsomal epoxide hydrolase to BaP-7,8-dihydrodiol. Further activation by CYP1A1 leads to BaP-7,8-dihydrodiol-9,10-epoxide (BPDE) which can form pre-mutagenic adducts by covalently binding to DNA (Arlt et al. 2008; Alexandrov et al. 2016; Kucab et al. 2019). BPDE mainly react with purine bases in DNA, with 10-(deoxyguanosin-*N*^2^-yl)-7,8,9-trihydroxy-7,8,9,10-tetrahydro-BaP (dG-*N*^2^-BPDE) being the most abundant DNA adduct detected in mammalian DNA (IARC 2012). Additionally, BaP can produce depurinating adducts by metabolite (e.g., *ortho*-quinones, benzylic sulphate esters, and radical cations) interactions with the N7 positions of purine bases which consequently lead to the generation of apurinic sites inside the sugar phosphate backbone of DNA (Rogan et al. 1993; Casale et al. 2001; Chiang and Means 2008). BaP can generate reactive oxygen species (ROS) indirectly (through the activity of redox metabolites) that can also damage the DNA by producing lesions (e.g., 9-OH- dG), propene adducts, or DNA single-strand breaks (Harvey et al. 2005). The BaP mediated genotoxic pathway has been studied in fine detail in higher eukaryotes and involves the intricate processing via CYP1 enzymes (CYP1A1, CYP1B1, and CYP1A2) (Shimada et al. 1997; Gotoh 1998; Xue and Warshawsky 2005; Shimada 2006; Luch and Baird 2010). There is evidence that members of the less-known CYP2 sub- family (e.g., CYP2C8, CYP2C9, CYP2C18, and CYP2C19) are also capable of metabolizing BaP (Guengerich and Shimada 1992; Bauer et al. 1995; Šulc et al. 2016). Indeed, many studies have demonstrated that the human exposure to PAHs (including BaP) results in the formation of DNA adducts (Albert et al. 1991; Harvey et al. 2005; Reed et al. 2018; Willis et al. 2018). A widely used and efficient technique in identifying DNA adducts induced by BaP (mainly the dG-*N*^2^-BPDE) is the thin-layer chromatography ^32^P-postlabelling (Phillips and Arlt 2007, 2020).

*Caenorhabditis elegans* is a non-parasitic nematode which lives within the interstitial water in the soil. Characterised by a fast generation time and large brood size (Riddle et al. 1997) and its completely sequenced and fully annotated genome, *C. elegans* has been subjected to many genetical and biochemical studies (Steinberg et al. 2008). Some 60–80% of genes within the worm genome are orthologous to their human counterparts (Lai et al. 2000; Kaletta and Hengartner 2006), for example 12 out of 17 signal transduction pathways are conserved between humans and *C. elegans* (NRC 2000). More importantly, 40% of human disease-associated genes are represented by orthologs in the *C. elegans* genome (Culetto 2000). *C. elegans* has been used in the discovery of pharmacological targets for human diseases and is gaining attention as a promising multicellular alternative for studying environmental pollutants at the molecular level as well as the level of whole organisms (Meier et al. 2014; Polak et al. 2014; Steinberg et al. 2008; Volkova et al. 2020). Previous toxicological research with *C. elegans* mainly focused on inorganic substances, such as heavy metals (Williams and Dusenbery 1990; Harada et al. 2007) or pesticides (Jones et al. 1996; Rajini et al. 2008). Only few studies have centred around PAHs or more specifically BaP. Life cycle assessment revealed that growth, reproduction and survival are impacted in *C. elegans* after BaP exposure (Sese et al. 2009). DNA damage, measured as DNA breaks, was also observed in BaP-exposed worms when using the alkaline version of the single cell electrophoresis assay (comet assay) (Imanikia et al. 2016).

The present study investigated phenotypical alterations, molecular genetic responses, and genotoxicity induction of nematodes exposed to the environmental carcinogen BaP. The *C. elegans* model is uniquely positioned as it lacks the classical CYP1A1 pathway and thus allows the investigation of alternative mechanisms that drive BaP (geno)toxicity.

## Materials and methods

### Chemicals

BaP (CAS number 50-32-8; purity ≥ 96%; Sigma-Aldrich, USA) was dissolved in dimethyl sulfoxide (DMSO; Sigma-Aldrich) to make a 40 mM stock solution.

### *C. elegans* strains and maintenance

*C. elegans* strains were maintained at 20 °C on Nematode Growth Media (NGM) plates supplemented with *Escherichia coli* OP50 as the food source. The *C. elegans* wild-type N2 Bristol as well as the mutant strains RB1788 [genotype: C03G6.14, *cyp-35A1* (*ok2306*) V.], VC743 [genotype: C03G6.15, *cyp-35A2* (*gk326*) V.], RB2046 [genotype: K09D9.2, *cyp-35A3* (*ok2709*) V.], RB1613 [genotype: K07C6.5, *cyp-35A5* (*ok1985*) V.] were obtained from the Caenorhabditis Genetics Center (CGC), University of Minnesota. For each assay, nematodes were age-synchronised using an alkaline hypochlorite treatment to isolate the eggs. Thereafter, eggs were allowed to hatch overnight in M9 solution and arrested at L1 stage. On the following day, age synchronous L1 worms were transferred to NGM plates and utilised for the assays.

### Exposure of *C. elegans* to BaP

BaP exposures were achieved by mixing *E. coli* OP50 bacteria with the appropriate volume of BaP stock solution to reach the desired concentration (0–40 µM) for testing. DMSO at a final concentration of 0.1% was used throughout (including untreated controls). Aliquots of bacteria and BaP mixture were seeded on NGM plates and incubated at room temperature for 2 days. Thereafter, L1-synchronised worms were plated on 90 mm petri dishes which contained the bacteria (a culture containing BaP or DMSO [controls]). Worms were incubated at 20 °C for 48 h to reach the L4 stage.

### Reproduction assay

Twenty-four age-synchronised L4 nematodes grown on OP50-inoculated NGM plates, supplemented with different concentrations of BaP, were individually transferred to 12-well OP50-inoculated NGM plates supplemented with corresponding BaP concentrations. Worms were transferred daily to the same well positions of new 12-well plates under the same conditions until egg laying ceased, typically within a 4-day period. One day after the eggs hatched, images of the individual wells were taken using a high-resolution camera, and the number of viable L1 larvae were counted daily over a 6-day period.

### Life span assay

Age-synchronised L1 worms were seeded onto OP50-inoculated NGM plates supplemented with different BaP concentrations. After 2 days, when the worms reached L4 stage, 200–400 worms were transferred to new plates containing the same BaP concentration. After that, daily transfers of all worms were performed. The worm status was assessed while transferring with a sterile platinum wire, scoring the worms as alive, dead, or lost (censored). The data input was analysed and processed in GraphPad Prism (version 8.2.1) by the Kaplan–Meier method, where the median survival, in days, were compared.

### Comet assay

The basic procedure described in this study is adopted from Imanikia et al. (2016). Modifications were made to increase the sensitivity and specificity by applying formamidopyrimidine DNA glycosylase (FPG) which is a base excision repair enzyme able to recognize and remove a wide range of oxidised purines from corresponding damaged DNA. After lysis of the cells, the microscope slides were incubated twice in FPG-buffer for 10 min. Therefore, 45 μL of 10,000 times diluted FPG enzyme (Sigma-Aldrich) was introduced to each slide and incubated in a humidity chamber at 37 °C for 30 min. Comets were analysed using a Leica fluorescence microscope (Leica DMLB 020-519-010 LB30T). DNA damage was scored using the Comet IV capture system (version 4.11; Perceptive Instruments, UK). Each technical replicate consisted of three slides in which fifty cell nucleoids were assessed per slide, and each sample was analysed in triplicate. All samples were scored blind. The tail intensity (% tail DNA), defined as the percentage of DNA migrated from the head of the comet into the tail, was used as a measure of DNA damage induced.

### Genomic DNA extraction and ^32^P-postlabelling assay

Age-synchronised L4 worms, grown under different BaP concentrations, were collected from plates with M9 buffer, serially washed with M9 buffer, mixed with sterile glass beads, and flash-frozen in liquid nitrogen. A 400 μL mixture of ethylenediaminetetraacetic acid (EDTA, 1 mM) and tris(hydroxymethyl)aminomethane (Tris, 50 mM) was added to each tube before vertexing for 5 min. Then, the supernatants were transferred to new 1.5-mL microcentrifuge tubes, and 9 μL of an RNase mixture of equal amounts of pancreatic ribonuclease (RNase A, 10 mg/mL, Sigma-Aldrich) and ribonuclease T1 (RNase T1, 50 KU, Sigma-Aldrich) were added to the samples. The tubes were incubated on a shaker (400 rpm) at 37 °C for 30 min. Thereafter, 40 μL of a freshly prepared proteinase K [10 mg/mL dissolved in a mix of EDTA (1 mM) and Tris (50 mM)] were added and mixed with the samples. The tubes were incubated on a shaker (400 rpm) at 37 °C overnight. The following day, DNA was isolated using a standard phenol–chloroform extraction method. DNA pellet was resuspended in Tris-EDTA (TE) buffer were stored at − 20 °C until analysis. The presence of BaP-derived DNA adducts (dG-*N*^*2*^-BPDE) was assessed using the nuclease P1 enrichment method of the ^32^P-postlabelling protocol as described previously (Arlt et al. 2008; Phillips and Arlt 2020).

### Total RNA extraction

A minimum of 7000 synchronised L4 stage *C. elegans* were washed off from NGM plates and collected. Total RNA was extracted using Tri-reagent (Sigma-Aldrich, St. Louis, MO, USA) modified to include a homogenization of nematodes by vortexing with an equal quantity of acid-washed glass beads (particle size 425–600 μm, Sigma-Aldrich). The concentration and integrity of total RNA was determined with a NanoDrop 1000 Spectrophotometer (NanoDrop Technologies, Inc., Wilmington, DE, USA) and by 2% agarose gel electrophoresis.

### RNA sequencing and data processing

RNA was extracted from BaP-exposed L4 nematodes, frozen at − 80 °C. The RNA integrity number (RIN) for each sample was determined via the Agilent Technology 2100 Bioanalyzer system using an RNA 6000 Nano kit (Supplementary figures 1 and 2). RNA-seq libraries prepared by GENEWIZ™ (Essex, UK) according to Illumina's instructions. Briefly, mRNAs were purified using Poly(A) selection from total RNA and then fragmented. First strand of cDNA was synthesised using random priming, followed by the synthesis of the second strand of cDNA. The resulting double-strand cDNA was end repaired, phosphorylated and A-tailed. Adapter ligation and PCR amplification were performed, rendering the library ready for Illumina flow cell clustering and sequencing on an Illumina HiSeq 2500 to sequence tens of millions of sequence clusters in parallel (Bentley et al. 2008).

Sequence reads (at least 30 million per sample/replicate) were trimmed to remove possible adapter sequences, sequence reads shorter than 30 nucleotides and nucleotides with poor quality (error rate < 0.05) were removed. The sequence reads were mapped to the *C. elegans* reference genome using the CLC Genomics Server program and the hit counts and RPKM values for genes calculated. After quantile normalization and log2-transformation on RKPM values, unsupervised hierarchical clustering and Principal Component Analysis (PCA) were performed. Comparisons of genes between various groups of samples were performed. A student *t* test was conducted for each comparison after quantile normalization and log2-transformation. A gene was selected if the *p* value was < 0.05 and the fold-change of normalized RPKMs was > 2. To analyse the variation of gene expression across the duplicates, the mean normalized RPKMs, standard deviation and coefficient of variation (CV) was calculated within each group.

### Real-time quantitative PCR

cDNA was synthesized from 1000 ng RNA using an oligo dT primer (5′-(T)_20_VN-3′) and M-MLV reverse transcriptase (Promega, Southampton, UK) applying standard incubation conditions. The transcript quantity was measured on an ABI Prism 7500 Fast (Applied Biosystems®, Paisley, UK) using the housekeeping gene *rla-1* (acidic ribosomal subunit protein P1) for normalization purposes (Swain et al. 2004; Polak et al. 2014). All probes and primers were designed to be compatible with the Universal Probe Library (Roche Applied Sciences, Burgess Hill, UK) (Supplementary 3). The CT values were determined using the 7500 Fast System SDS Software (Applied Biosystems®) and the fold changes in gene expression were calculated by applying the 2^−ΔΔCt^ method. Statistical analysis was performed on three independent biological replicates, each consisting of three technical repeat measurements.

### Statistical analysis

The analysis of variance (ANOVA) was employed to evaluate the majority of data in the study. *T* tests were employed to scrutinize the variation significance between an exposure sample and a control. All statistical tests for this project were conducted using GraphPad Prism (version 8.2.1). Statistical significances throughout were indicated by asterisks; * for a *p* value ≤ 0.05, ** for a *p* value ≤ 0.01, *** for a *p* value ≤ 0.001, and **** for a *p* value ≤ 0.0001.

## Results

### Impact of BaP exposure on reproduction

**Fig. 1:**
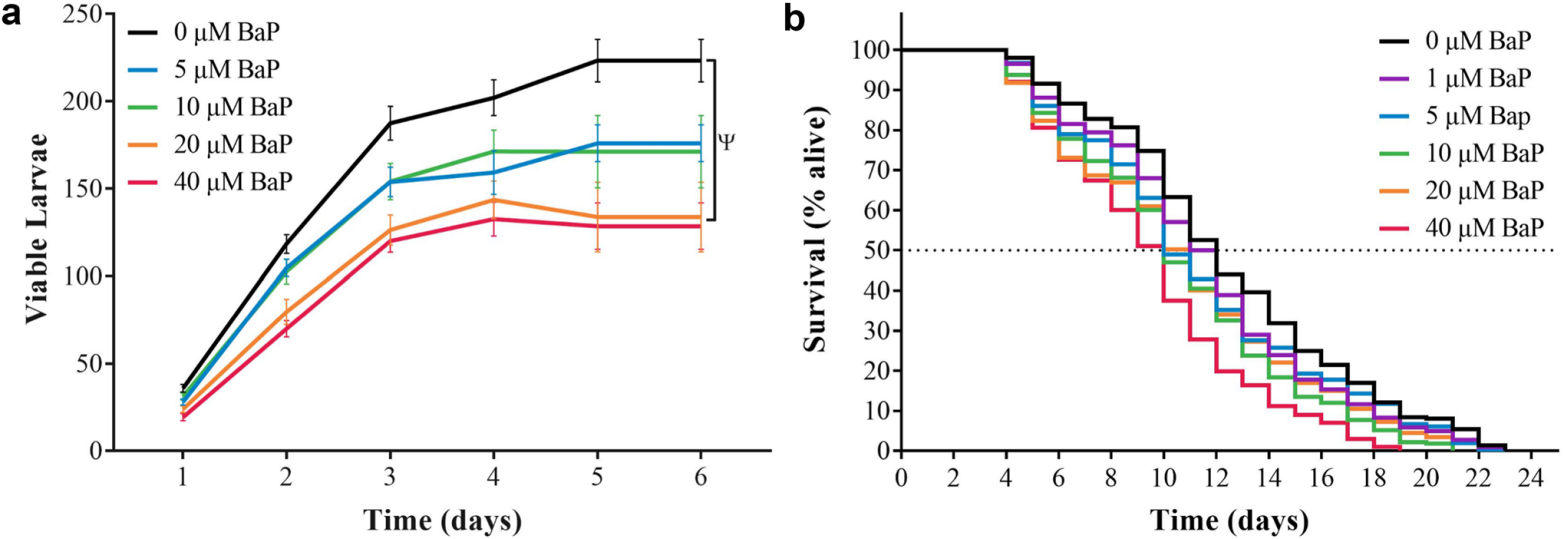
**a** Wild-type *C. elegans* were exposed to BaP (0–40 µM) for 8 days, and the average daily number of viable larvae was counted every 24 h and cumulatively added during the egg laying phase (marked 1–6). Error bars represent SEM. Statistical analysis was performed using a two-way ANOVA, followed by a Tukey’s multiple comparisons test, *n* = 24 per BaP concentration. *Ψ* = the *p* value of 0 vs. 20 µM BaP ≤ 0.01, and of 0 vs. 40 ≤ 0.001. **b** The percentage survival of wild-type *C. elegans* exposed to different concentrations of BaP (0–40 μM). The worms were scored by transferring them all to a new plate every 24 h from L1 stage until all worms were dead. Lost or mistakenly killed worms were censored and removed from the data. Note that the median survival (50% alive) of worms was measured to be 12 days for the control worms (BaP [0 μM]; DMSO [0.1% v/v]) but only 10 days for the highest BaP concentration (40 μM). Statistical analysis was performed using log-rank (Mantel-Cox) test, *n* = 400 per BaP concentration. All samples contained DMSO (0.1% v/v)

A key endpoint which evaluates toxicity of xenobiotics in *C. elegans* is brood size*.* Changes in reproductive output in BaP-exposed wild-type worms (0–40 μM from L1 to L4 stage) was assessed by counting the number of viable offspring during the egg laying period. The cumulative brood size was determined to be 223 ± 12 viable larvae in wild-type control (unexposed). In comparison, the cumulative average number of viable larvae decreased significantly with increasing BaP concentration, resulting in 176 ± 11 and 171 ± 21 offspring per worm exposed to 5 and 10 μM, respectively. For worms exposed to 20 μM BaP, the reproductive output decreased to 134 ± 20. Exposure to 40 μM BaP for 6 days reduced the number of viable larvae to 129 ± 13 (Fig. 1a). During the first 3 days of egg laying, a significant decrease in the number of the viable larvae was observed in worms exposed to 20 μM and 40 μM BaP in comparison with control (unexposed) worms.

### Impact of BaP exposure on life span

The life span assay is used routinely to determine the effect of a xenobiotic chemical on aging and death (Koch et al. 2014). Age-synchronised L1 wild-type *C. elegans* were exposed to different concentrations of BaP (0–40 μM), transferred daily onto freshly prepared NGM plates with the same experimental conditions and the numbers of alive, dead, and censored worms were recorded. This was repeated until all worms of all groups died. The median survival (50% alive) of wild-type control worms was determined to be 12 days which decreased by 1–2 days at concentrations of BaP above 1 μM BaP, an effect which was statistically significant (Fig. 1b).

### Induction of DNA strand breaks (comet assay) after BaP exposure

**Fig. 2:**
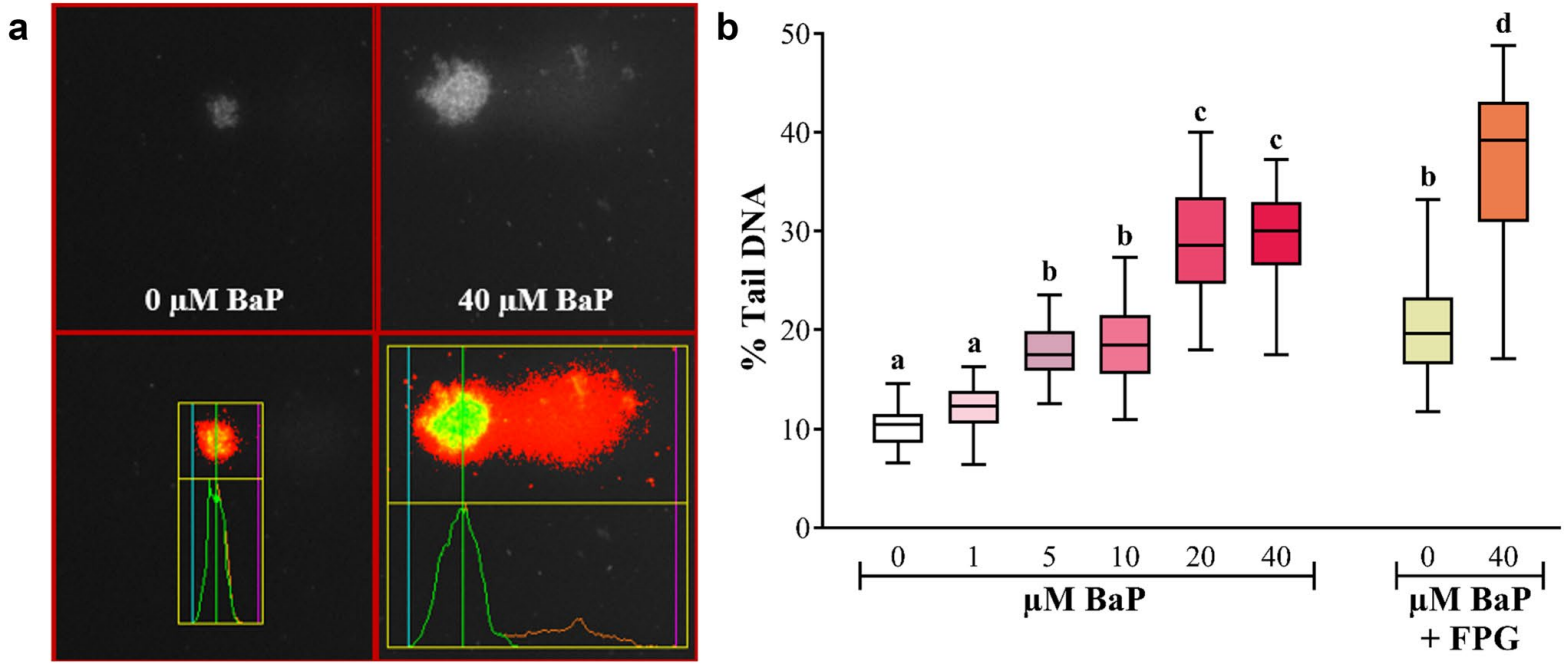
**a** Images on the upper row are representative cells after performing the comet assay isolated from wild-type *C. elegans* exposed for 48 h to different BaP doses (0, 1, 5, 10, 20, or 40 μM). The images on the lower row are outputs from the Comet IV software (version 4.11, Perceptive Instruments Ltd., UK). **b** A box plot of the % tail DNA in cells isolated from wild-type *C. elegans* exposed for 48 h to different doses of BaP (0, 1, 5, 10, 20, or 40 μM) and treatment with FPG at 0 and 40 μM BaP. Fifty cell nucleoids were measured per slide (technical replicates), for a total of 150 cells per biological replicate (3 slides) and 450 total cells per experimental condition (3 biological replicates). The average of 150 cells was calculated for all biological replicates and then averaged (*n* = 3). Statistical analysis was performed using a one-way ANOVA, followed by a Tukey’s multiple comparisons test; a, b, c, d refers to the calculated probability (*p* value), where different letter denote *p* ≤ 0.0001. All samples contained DMSO (0.1% v/v)

Wild-type nematodes were exposed to various concentrations of BaP (0, 1, 5, 10, 20, and 40 μM) for 48 h. Cells successfully isolated from these worms were then embedded onto pre-coated agarose slides. A portion of these slides was further incubated with FPG to measure oxidative damage to DNA. FPG is a base excision repair enzyme which recognises and removes a wide range of oxidised purines from the corresponding damaged DNA. This additional step increases the sensitivity and specificity of the comet assay (Hansen et al. 2010). Afterwards, all the slides were run through a single-cell gel electrophoresis (comet assay) and DNA damage was scored using the Comet IV capture system (Fig. 2a). No inter-sample differences in atypical comets (e.g., hedgehogs) were noted; however, an increase in the tail intensity (% tail DNA) was observed after exposure to increasing BaP concentrations. The baseline in control (unexposed) worms was about 10%. After BaP exposure tail intensity increased to ~ 17% in worms treated with 5 and 10 μM BaP and further increased up to ~ 30% after treatment with 20 and 40 μM BaP. When the FPG-modified comet assay was performed background DNA damage increased from 10 to 19% in controls (unexposed). Similarly, the % tail DNA generated from a comet assay on cells dissociated from nematodes exposed to 40 μM BaP also increased by ~ 9% when the comet assay was modified with FPG (from ~ 30% to ~ 39%) (Fig. 2b). No information was gathered regarding the viability of the cells used for the comet assay.

### DNA adduct formation after BaP exposure

**Fig. 3:**
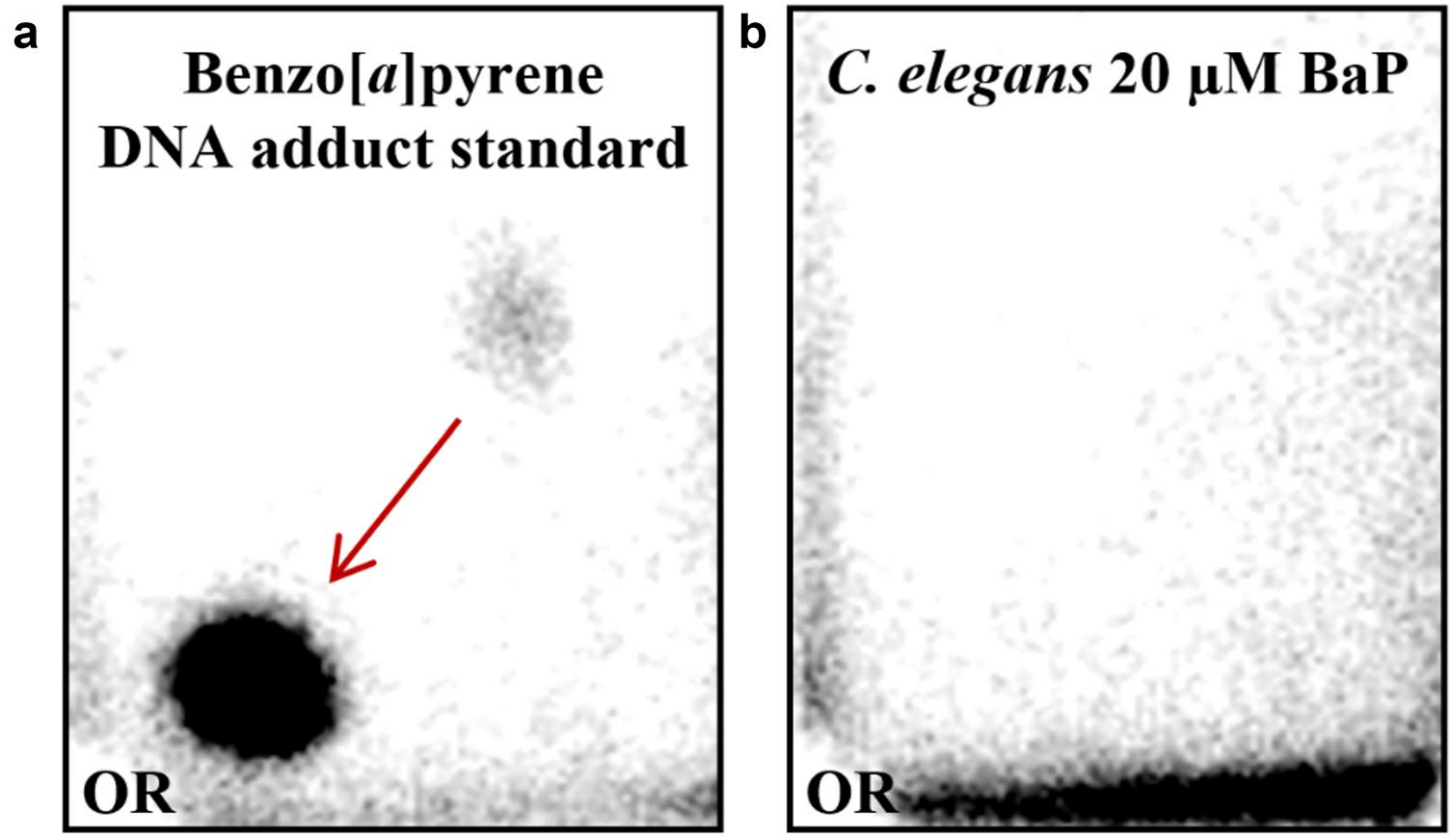
Representative autoradiographic profiles of DNA adducts obtained by TLC ^32^P-postlabelling in wild-type *C. elegans* exposed to 20 µM BaP for 48 h. **a** Liver DNA isolated from mice treated with a single intraperitoneal dose of 125 mg/kg body weight BaP was used as positive control (Arlt et al. 2012). **b** The arrow indicates the dG-*N*^2^-BPDE adduct. Solvent conditions for the separation of BaP-derived DNA adducts on PEI-cellulose TLC were as follows: D1, sodium phosphate (1 M), pH = 6.0; D3, lithium formate (3.5 M), urea (8.5 M), pH = 3.5; D4, lithium chloride (0.8 M), Tris (0.5 M), urea (8.5 M), pH = 8.0. The origins (OR), at the bottom left-hand corners, of each chromatogram were cut off before exposure

For DNA adduct analysis the ^32^P-postlabelling assay was performed (Phillips and Arlt 2020) which is capable of detecting BaP-derived DNA adducts (Arlt et al. 2008). As shown in Fig. 3a, using BaP-exposed mouse liver DNA as positive control, BaP exposure lead to one major DNA adduct spot on the thin-layer chromatography plate which was previously identified as dG-*N*^2^-BPDE (Arlt et al. 2008). In contrast, no BaP-derived DNA adducts (i.e., dG-*N*^2^-BPDE) were detected in wild-type *C. elegans* exposed to 20 μM (Fig. 3b) and 40 μM BaP for 48 h. Nematodes exposed to BaP were independently tested twice, Fig. 3 showing representative images.

### Transcriptional analysis

**Fig. 4:**
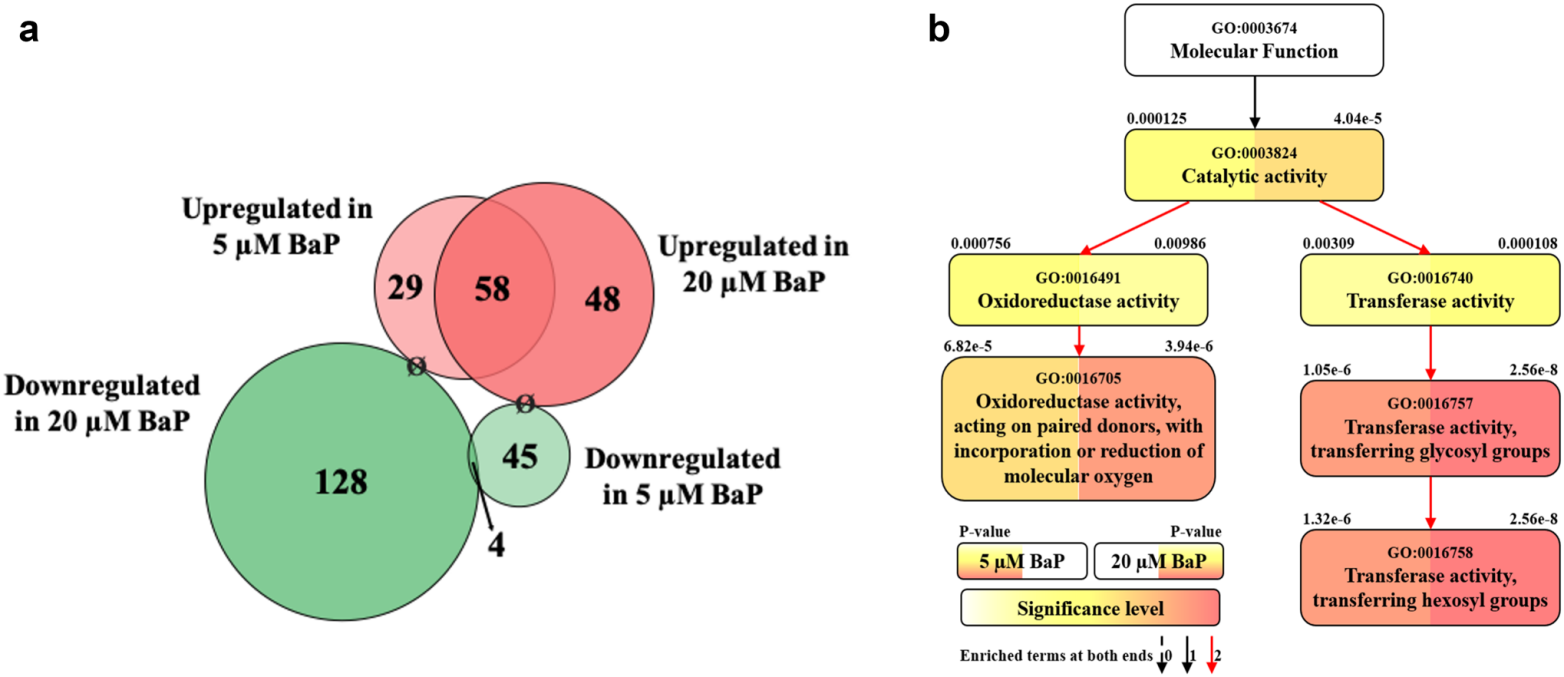
**a** Proportionally sized Venn diagram showing the 312 significantly (*t* test, *p* ≤ 0.05, *n* = 3) and differentially regulated genes (> 2-fold change) of wild-type *C. elegans* exposed to different doses of BaP (0, 5, or 20 µM) for 48 h. Ø marks a zero gene overlap. **b** Partial gene ontology (GO) hierarchical tree presenting molecular functions which were found to be significantly enriched in wild-type *C. elegans* exposed to different concentrations of BaP (0 vs. 5 and 0 vs. 20 µM) for 48 h. Boxes on the graph represent GO terms labelled with their GO number, term definition, and statistical information (*p* value) on both sides on top of the boxes (5 and 20 µM BaP on the left and right side, respectively). The degree of colour saturation (from white through yellow to red) of one side of the box is positively correlated to the enrichment level of the term for that exposure condition. Black dashed, black solid, and red solid lines represent zero, one, and two enriched terms at both ends connected by a line. The graph was based on the results of DAVID Bioinformatics Resources version 6.8 and PANTHER classification version 14.0 and was constructed manually. All samples contained DMSO (0.1% v/v) (colour figure online)

A global RNA-seq transcriptomic experiment was investigated to provide an understanding of the underlying mechanisms by which BaP exerts its effects on the nematode. Principal component analysis (PCA) was performed using the ClustVisTM web tool (Metsalu and Vilo 2015) which illustrates the reproducible technical generation of samples (Supplementary Figures 4 and 5). Additional validation on 15 transcripts was performed by qPCR which confirmed that the overall trend in expression dynamics was comparable between RNA-seq and qPCR (Supplementary Figures 6 and 7). A distinct transcriptional response was observed, namely the expression of only 312 transcripts were significantly (*p* ≤ 0.05) and differentially (2-fold or higher) altered after BaP exposure to either 5 or 20 μM BaP in comparison to control (unexposed) (Fig. 4a). Many of the most significantly up-regulated transcripts were common in worms exposed to 5 μM or 20 μM BaP, with clear dose-dependent increase in expression levels (Table 1). In contrast, only four of the 177 significantly (*p* ≤ 0.05) down-regulated transcripts were common in worms exposed to 5 or 20 μM BaP. No genes were up-regulated in samples exposed to 5 μM BaP and at the same time down-regulated in samples exposed to 20 μM BaP, nor vice versa (Fig. 4a).

**Table 1:**
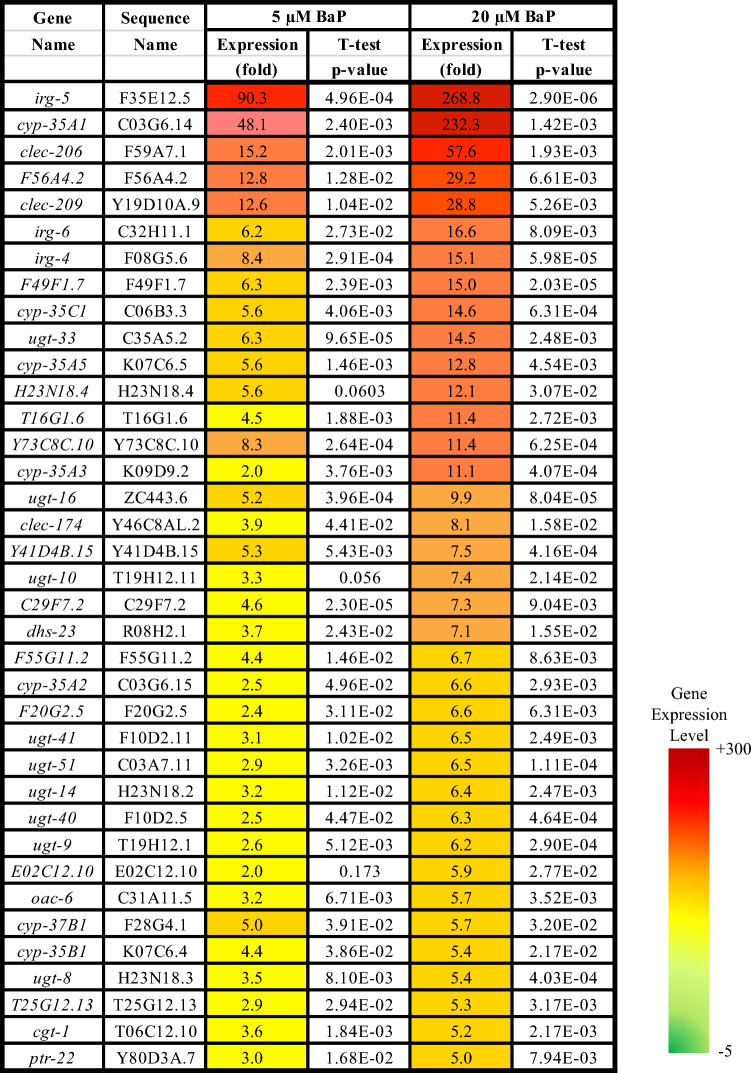
Top upregulated gene with their sequence names, their expression differences (0 vs. 5 and 20 µM BaP), and their *t* test *p* values obtained from the RNA-seq data (0 vs. 20 µM BaP, > 5-fold change, *t* test, *p* ≤ 0.05, *n* = 3) in comparison to the control (0 µM BaP)

Two main gene groups were significantly altered after BaP exposure. Firstly, the *cyp* genes (especially the *cyp-35* family), which are linked to xenobiotic response, redox reactions, and lipid metabolism. Seven (of the 10) *cyp-35* genes were represented in the list of the top 37 BaP most highly induced genes (0 vs. 20 μM, > 5-fold induction, *t* test, *p* ≤ 0.05, *n* = 3) (Table 2). Secondly, UDP-glucuronosyltransferase (UGT) genes, which are linked to the biotransformation of xenobiotics, including phase II metabolism of BaP in higher eukaryotes (Brand et al. 2010; Kurita et al. 2017). Nine (of 65) *ugt* genes were in the list of the top 37 BaP-induced genes (0 vs. 20 μM, > 5-fold induction, *t* test, *p* ≤ 0.05, *n* = 3) (Table 3).

**Table 2:**
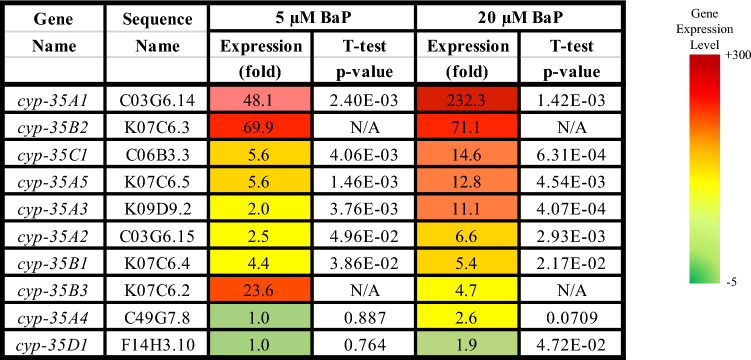
List of all *cyp-35* genes in wild-type *C. elegans* with their sequence names, their expression differences (0 vs. 5 and 0 vs. 20 µM BaP), and their *t* test *p* values as obtained from the RNA-seq data (*t* test, *p* ≤ 0.05). N/A = not available

**Table 3:**
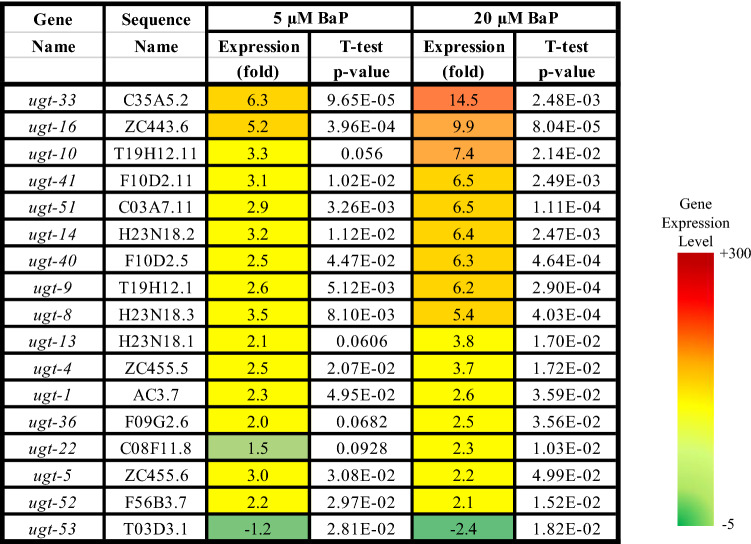
List of all significantly and differentially regulated *ugt* genes, out of a total of 65 *ugt* genes (> 2-fold change, *t* test, *p* ≤ 0.05, *n* = 3), with their sequence names, their expression differences (0 vs. 5 and 0 vs. 20 µM BaP), and their *t* test *p* values, as obtained from the RNA-seq data

In addition to genes linked to BaP metabolism, based on the RNA-seq, two further groups of genes were significantly and differentially regulated, namely infection response and innate immune response genes such as F35E12.5 (*irg-5)*, F08G5.6 (*irg-4*), C32H11.1 (*irg-6*) and C-type lectin (*clec*) genes. F35E12.5 (*irg-5*) was the most significantly up-regulated gene in the data set; it was induced ~ 90-fold at 5 μM BaP and ~ 270 fold at 20 μM BaP, respectively.

The most significant molecular functions (GO:0003674) obtained from the RNA-seq data set were the oxidoreductase activity, acting on paired donors, with incorporation or reduction of molecular oxygen (GO:0,016,705) and transferase activity, transferring hexosyl groups (GO:0016758) (Supplementary 8). These are linked to the significantly (*t* test, *p* ≤ 0.05) up-regulated *cyp* genes (mainly the *cyp- 35′s*) and the large number of the significantly (*t* test, *p* ≤ 0.05) up-regulated *ugt* genes, respectively (Fig. 4b).

### The CYP phylogenetic tree

**Fig. 5:**
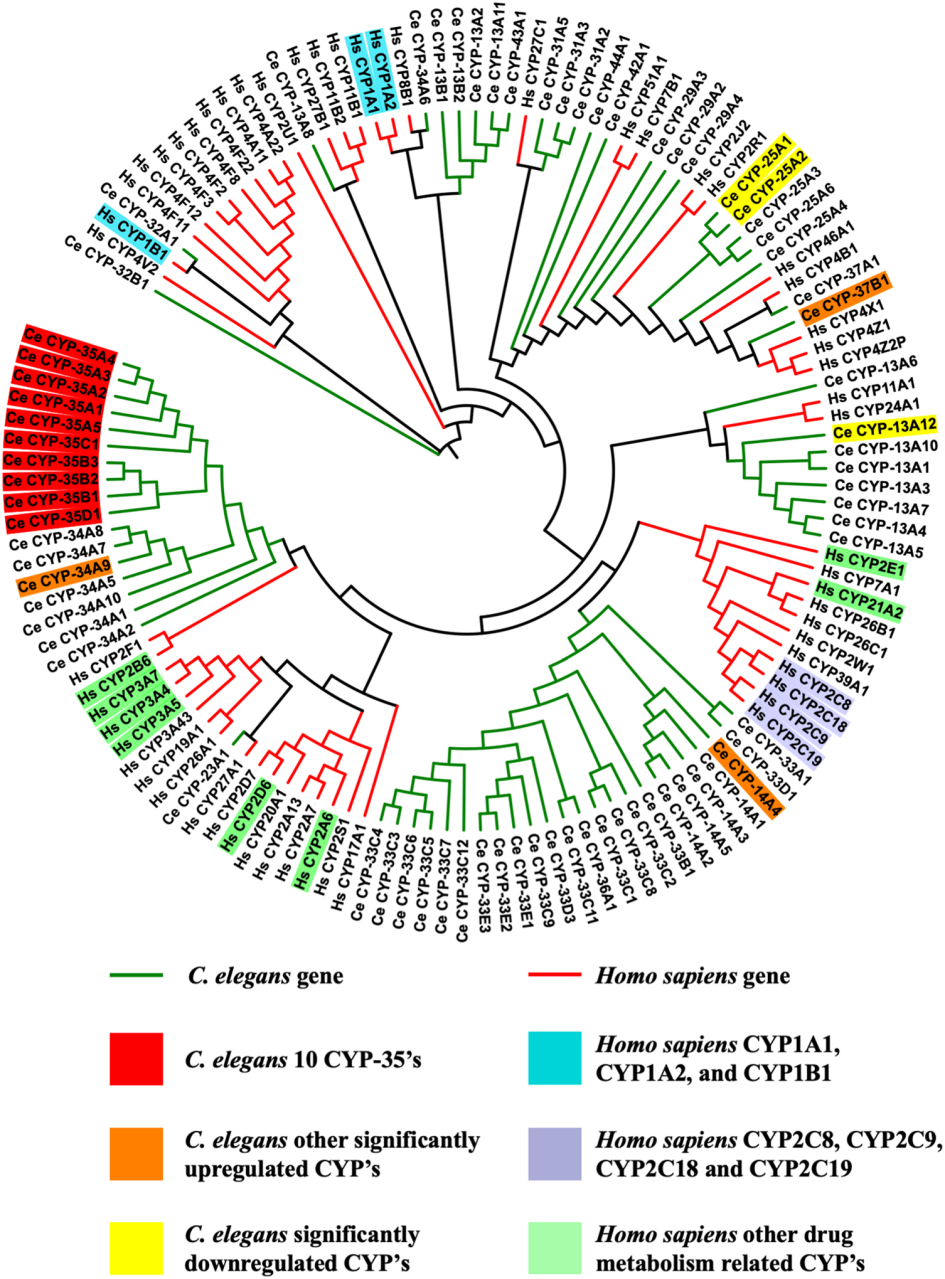
Maximum likelihood cladogram showing the relationships between the different CYP proteins of *C. elegans*: 10 CYP-35′s (red), other significantly (*t* test, *p* ≤ 0.05, *n* = 3) up-regulated CYP’s (orange), and the significantly (*t* test, *p* ≤ 0.05) down-regulated CYP’s (yellow), in *Homo sapiens*: CYP1′s (blue), the CYP2C’s (purple), and others (green). The tree was generated using the MEGA software, version 7.0.26 (colour figure online)

To investigate the relationship between human and *C. elegans* BaP metabolism, a phylogenetic tree was constructed consisting of the *Homo sapiens* CYPs implicated in the metabolism of BaP, CYP1A1, CYP1B1, and CYP1A2 (Shimada et al. 1997; Gotoh 1998; Xue and Warshawsky 2005; Shimada 2006; Luch and Baird 2010), CYP2s (Wang et al. 2017), and others (Prakash et al. 2015) as well as the 75 CYPs expressed in *C. elegans*. Based on sequence homology, the majority of the human and worm CYPs clustered, at large, distinctly separate, with the human CYP1s / CYP2Cs and the worm CYP-35′s positioned within separate clades (Fig. 5). The *H. sapiens* CYP2Cs are located in neighbouring clades close to *C. elegans* CYP-33 s and CYP-14 s, with *cyp-14A4* notably shown to be significant upregulated in worms exposed to BaP. *C. elegans* CYP-34A9, the other upregulated CYP in worms, is also positioned close to the CYP-35s family. An equivalent phylogenetic tree was generated for UGTs, again suggesting that worm and mammalian UGT’s differ, at least by sequence homology (Supplementary 9).

### The effect of BaP on knockout mutant strains

**Fig. 6:**
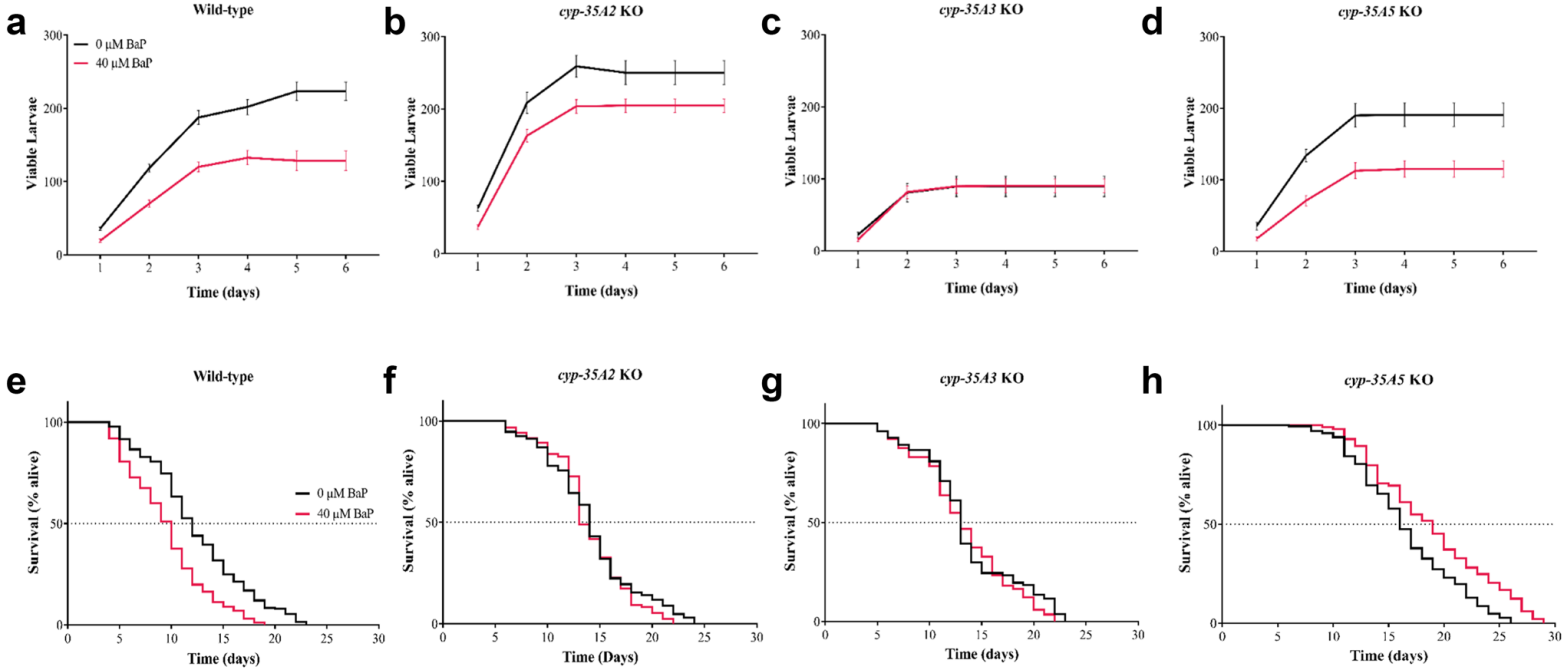
Brood size and percentage survival of wild-type *C. elegans* and *cyp-35* knockout (KO) strains that were exposed to BaP (0 and 40 µM). **a–d** Wild-type *C. elegans* and *cyp-35* knockout (KO) strains were exposed to BaP (0 and 40 µM) for 8 days, and the average daily number of viable larvae was counted and cumulatively added during the egg laying phase, i.e., 6 days (starting at day 4 from L1) and are labelled 1–6 on the graphs. The number of viable larvae was counted every 24 h. Error bars represent SEM. Statistical analysis was performed using a two-way ANOVA, followed by a Sidak’s multiple comparisons test, *n* = 24 per condition. **e–h** The worms were scored every 24 h until all worms were dead. Statistical analysis was performed using the log-rank (Mantel-Cox) test, *n* = 200 per BaP concentration per strain. All BaP doses contained DMSO (0.1% v/v)

Five *cyp-35* genes (*cyp-35A1*, *cyp-35A2*, *cyp-35A3*, *cyp-35A5*, and *cyp-35B1*), identified by RNA-Seq as key genes with possible links to xenobiotic exposure and metabolism, were selected to explore their involvement on BaP mediated changes in the physiological end points measurements. When *cyp-35A2* KO worms were exposed to 40 μM BaP, their average cumulative viable larvae count was significantly higher compared to wild-type nematodes exposed to the same BaP concentration (Fig. 6a, b). In addition, there was no significant change in the average cumulative number of viable larvae between control (unexposed) and BaP-exposed *cyp-35A2* KO nematodes (Fig. 6a, b). The average median survival of *cyp-35A2* KO worms exposed to 40 μM BaP did not significantly change in comparison to controls (unexposed) (Fig. 6f). However, the median survival of *cyp-35A2* KO worms was significantly longer than wild-type nematodes after exposure to 40 μM BaP (Fig. 6e, f).

The average number of offspring produced by *cyp-35A3* KO worms to BaP was significantly lower compared to wild-type nematodes in the presence or absence of BaP exposure (Fig. 6a, c). On the other hand, there was no significant change in the average cumulative number of viable larvae between the control (unexposed) and BaP-exposed *cyp-35A3* KO nematodes (Fig. 6c). Also, the median survival did not differ in *cyp-35A3* KO nematodes after exposure to 0 or 40 μM BaP (Fig. 6g); however, compared to wild-type nematodes their median survival after exposure to 40 μM BaP was significantly extended (Fig. 6e, g).

**Fig. 7:**
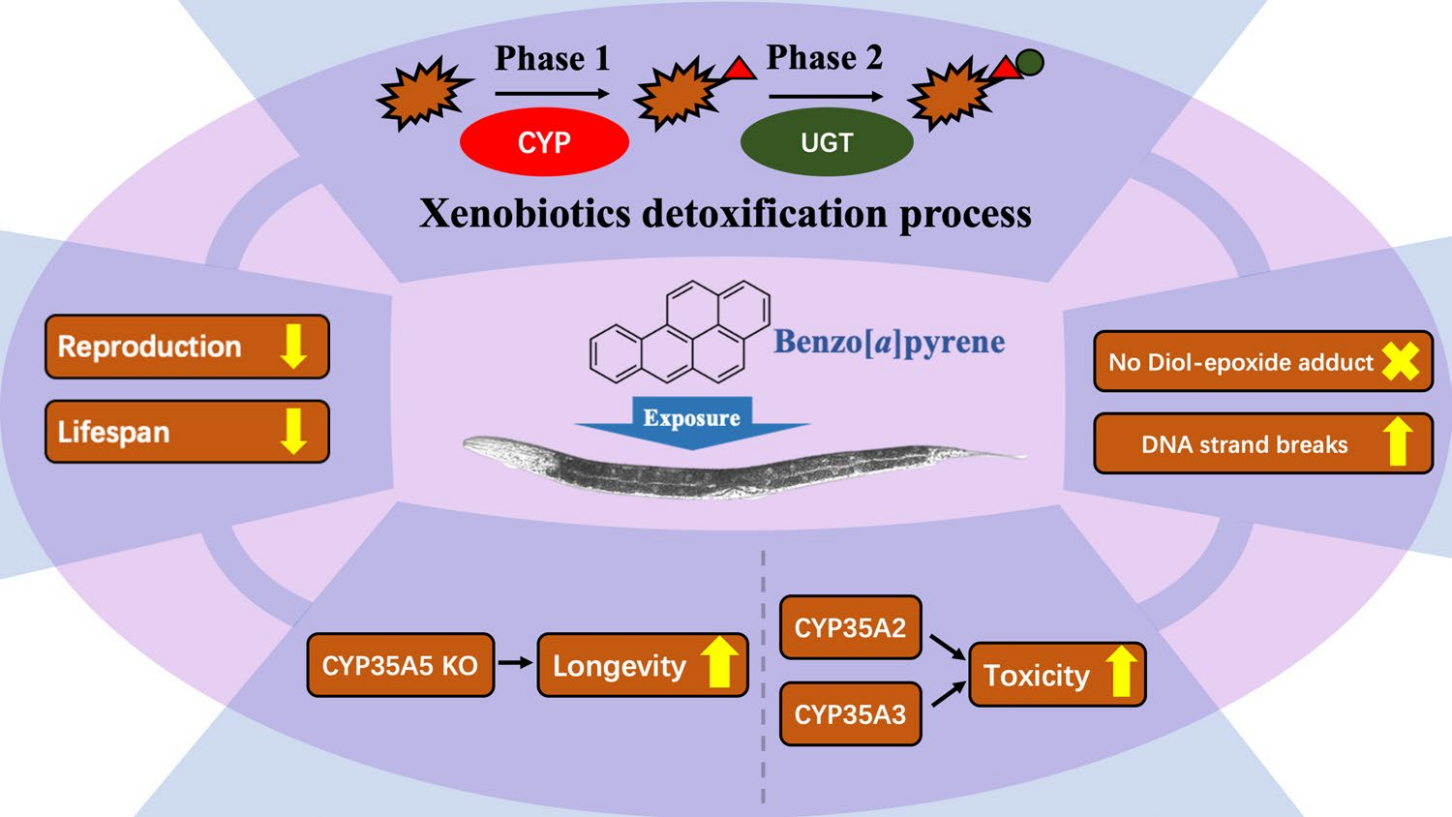
Summary of the molecular genetic and physiological responses of *C. elegans* exposed to BaP based on our research. The physiological end points of the wild-type animals showed the significant reduction of reproduction and lifespan. The global transcriptomic analysis demonstrated that the *cyp* and *ugt* families were involved in xenobiotics detoxification process. The assessment of genotoxicity confirmed an increase in DNA damage (comet) although no BaP-derived DNA adducts (i.e., dG-*N*^2^-BPDE) were detectable by ^32^P-postlabelling. The physiological measurements on KO strains revealed potential candidates which induce BaP toxicity and contribute to longevity

The average cumulative reproductive performance of *cyp-35A5* worms exposed to BaP did not significantly differ to wild-type nematodes (Fig. 6a, d**)**. The median survival of BaP-exposed *cyp-35A5* KO nematodes was, however, significantly extended compared to controls (unexposed) (Fig. 6h), which was also significantly extended compared to wild-type nematodes (Fig. 6e, h). Due to the internal hatching of the progeny, all nematodes of the *cyp-35A1* KO strain were dead by day 4 (Supplementary 10) (Fig. 7).

## Discussion

### Physiological endpoints in *C. elegans* an organism which lacks the classical CYP1 enzymes

The effects on key physiological endpoints underlines the notion that BaP induces substantial toxicological effects on *C. elegans* despite the fact that it lacks the CYP1 enzymes. Previous research focusing on the toxicity effects of BaP on *C. elegans* used the aqueous media (Sese et al. 2009; Ura et al. 2002; Haegerbaeumer et al. 2018). Our plate-based dosing method also revealed the significant reduction in reproductive capacity and life span observed in *C. elegans* exposed to BaP, and therefore, this aligns, in general, with previous reports.

### Genotoxicity in *C. elegans*

The existence of DNA strand breaks indicates the presence of a genotoxic potential. The alkaline single-cell electrophoresis, conventionally referred to as the alkaline comet assay, was utilised on cells dissociated from wild-type *C. elegans* exposed to BaP. The comet assay displayed a ~ 3-fold increased DNA damage in nematodes exposed to 40 μM BaP, a response which was similar to the data reported previously in our laboratory (Imanikia et al. 2016). Others have demonstrated that the comet assay can be modified with FPG which increases the sensitivity and specificity in mammalian systems (Speit et al. 2004), and the results obtained here suggested FPG addition improved the sensitivity of the comet assay but were inconclusive regarding BaP’s link to oxidative stress. The DNA damage identified here contradict the results of a previous investigation which didn’t detect DNA lesions of BaP exposed nematodes using a qPCR assay in aqueous media (Leung et al. 2010).

Interestingly, the ^32^P-postlabelling technique was not able to detect the presence of dG-*N*^2^-BPDE adducts in wild-type *C. elegans* exposed to BaP. Although the radical cation (Cavalieri and Rogan 1985; Devanesan et al. 1996) and o-quinone pathways (Penning 2014) for BaP lead to radical-DNA interaction and DNA depurination but their role in BaP carcinogenesis in *C. elegans* was beyond the scope of the present study and other techniques such as mass-spectrometry might be required to explore whether different forms of BaP metabolites generate other types of DNA adducts.

### Transcriptional analysis of *C. elegans* exposed to BaP

RNA-seq is a high-throughput assay which enabled the identification of transcriptomic changes in response to BaP exposure. The assay revealed that the 312 highly responsive transcripts were dominated by the *cyp-35′s* and *ugt’s* families with subsequent gene ontology analysis highlighting oxidation–reduction processes (GO:0055114), transferase activity (GO:0016758), and response to xenobiotic stimulus (GO:0009410), among others. The *cyp-35*s are thought to be part of the first stage xenobiotic detoxification response in *C. elegans* and were shown to be induced by β-naphthoflavone, PCB52, atrazine and lansoprazole. (Menzel et al. 2001, 2005, 2007; Lindblom and Dodd 2006; Harlow et al. 2018). BaP is metabolised in *Homo sapiens* mainly by the action of CYP1′s (CYP1A1, CYP1A2, and CYP1B1) (Shimada et al. 1997; Gotoh 1998; Xue and Warshawsky 2005; Shimada 2006; Luch and Baird 2010). However, members of the less-known CYP2 sub- family have been shown to metabolise BaP as well, namely CYP2C8, CYP2C9, CYP2C18, and CYP2C19 (Guengerich and Shimada 1992; Bauer et al. 1995; Šulc et al. 2016). However, inspection of the maximum likelihood tree derived from *C. elegans* and human CYP protein revealed no close CYP1 and CYP2C sequence homologs in the nematode. Besides, the protein sequence alignment between CYP-35A1 (P04798) and CYP1A1 (O02627) by Clustal Omega revealed an identity score of only 21%. The CYP-35s protein structure is at this point not unavailable, and although the structure of human CYP2A6 (PDB ID: 2FDV) is available, the sequence shares an identity of only 25% sequence with *C.elegans* CYP-35A1. To what extent the nematode CYP-35s can compensate or substitute the function of human CYP1 and CYP2C is at present not known.

Phase 2 metabolism comprises the detoxification reactions of xenobiotic metabolism, and UGT enzymes are a key player in this process. There are at least 72 genes that code for UGT-like proteins in *C. elegans* (Lindblom and Dodd 2006), 16 of which were shown to be up-regulated by BaP exposure. In humans, UGTs have been identified to play a role in the BaP detoxification process, which implies that nematode UGTs may have a similar function (Dellinger et al. 2006; Zhang et al. 2013a; Vergara et al. 2020).

### Physiological endpoints in mutant *C. elegans*

Five *C. elegans cyp-35* knock-out strains were selected and changes in the physiological end points evaluated upon exposure to BaP. Due to worm bagging of *cyp-35A1* KO nematodes, the brood size data as well as its life span data were only available for 4 days which rendered them unsuitable to investigate their role in BaP metabolism. *C. elegans* lacking *cyp-35A3* were characterised by an absence of a BaP-induced increase in toxicity, suggesting that *cyp-35A3* might play an important role in BaP metabolism. Previous reports have linked *cyp-35A1* and *cyp-35A3* to lipid metabolism (Aarnio et al. 2011; Zhang et al. 2013a, b; Imanikia et al. 2015), indeed, animals lacking another fat metabolising gene (*fat-3*) displayed a similar abnormal egg-laying behaviour (Lesa et al. 2003; Reisner et al. 2011). The nematodes with a *cyp-35A2* or *cyp-35A3* mutation exhibited no alteration in median life span after BaP exposure. Taken together this suggests that *cyp-35A1, cyp-35A2* and *cyp-35A3* are required to initiate the toxic effects of BaP.

BaP even extended the life span of *cyp-35A5* deletion mutant, which is the first report of its involvement in longevity in *C. elegans*. Previous studies demonstrated that the RNAi of *cyp-35A5* resulted in a lower fat content phenotype (Aarnio et al. 2011). Imanikia et al. (2016) stated that the expression of *cyp-35A5* and *daf-16* were upregulated in *fat-5;cyp-35A2* double thereby providing a tentative link between these two genes, where *daf-16* serves important stress responsive functions and regulate longevity (Barsyte et al. 2001; Garsin et al. 2003; Mendenhall et al. 2006; Shore and Ruvkun 2013). Furthermore, life span was prolonged by an additional 3 days when the *cyp-35A5* mutant was exposed to BaP. This extended longevity might be triggered by the activation of alternative CYPs which contributes to longevity through the clearance of toxins generated by endogenous processes, such as metabolism as well as lipophilic by-products (Gems and McElwee 2005).

## Conclusion and future perspectives

Overall, this study demonstrates that the exposure to BaP significantly affects *C. elegans,* including the physiological, genotoxic and transcriptional levels, notably despite the absence of a CYP1 homolog. The highly responsive *cyp-35* genes play a central role in regulating the BaP biotransformation response and appear to be a key player in the hitherto uncharacterised metabolic pathway of BaP in *C. elegans*. It is suggested that further genotoxic assays are performed and *C.elegans* CYP-35 antibodies raised to pinpoint the specificity and interplay of the *cyp-35* family in the worm’s response to BaP.

## Supplementary Information

Below is the link to the electronic supplementary material.

Supplementary material 1 (PPTX 1981 kb)

## Data Availability

The datasets generated during and/or analyses during the current study are available via GEO accession number GSE152257.
