# Supplementary material for: Benzo[a]pyrene and Caenorhabditis elegans: defining the genotoxic potential in an organism lacking the classical CYP1A1 pathway
Source: Arch Toxicol. 2021 Jan 9;95(3):1055–69. doi: 10.1007/s00204-020-02968-z (PMC7904753; doi:10.1007/s00204-020-02968-z)
Supplement: Supplementary file 1 — Supplementary material 1 (PPTX 1981 kb) [file 204_2020_2968_MOESM1_ESM.pptx]

## Slide 1
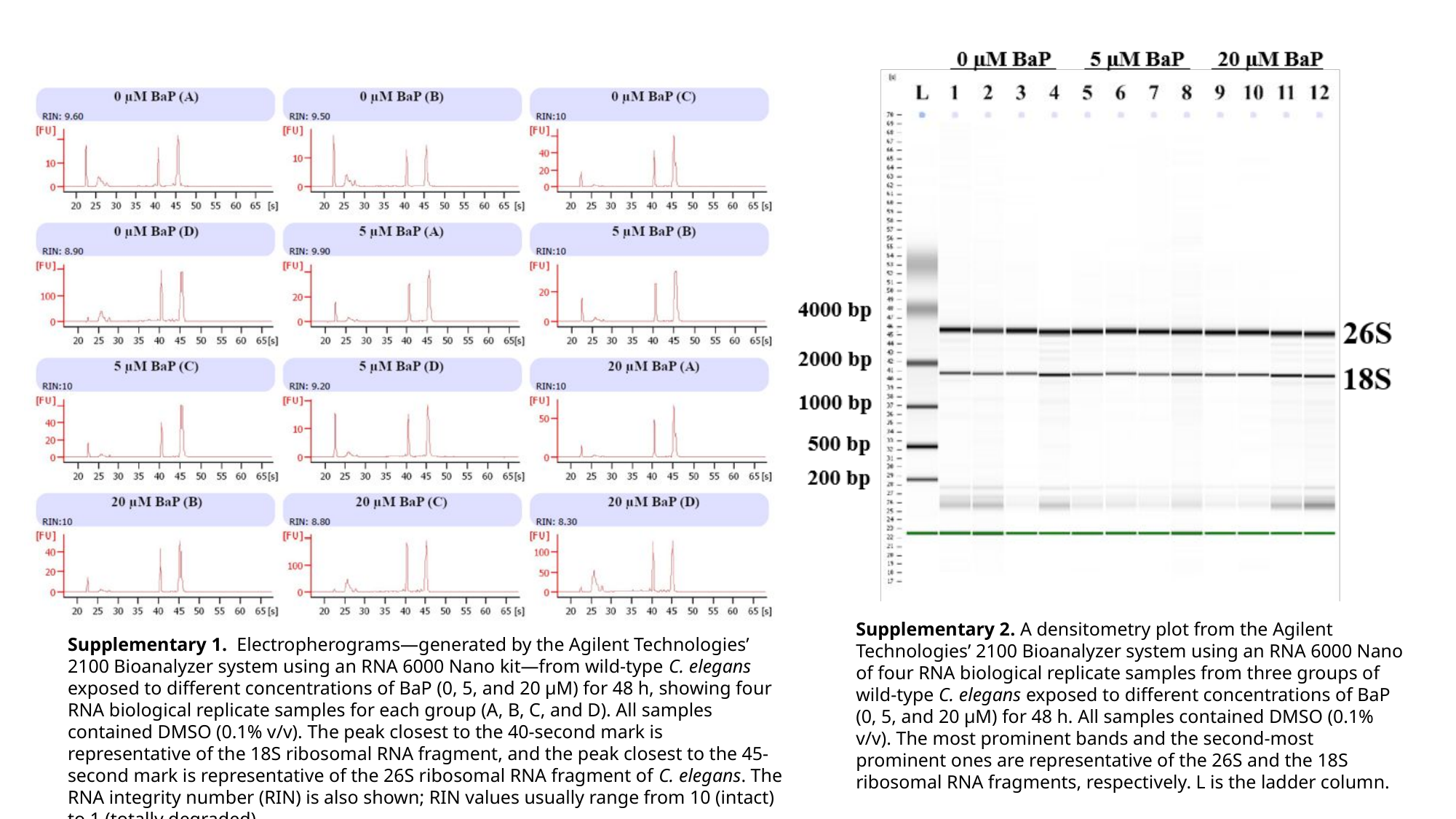

Supplementary 2. A densitometry plot from the Agilent Technologies’ 2100 Bioanalyzer system using an RNA 6000 Nano of four RNA biological replicate samples from three groups of wild-type C. elegans exposed to different concentrations of BaP (0, 5, and 20 μM) for 48 h. All samples contained DMSO (0.1% v/v). The most prominent bands and the second-most prominent ones are representative of the 26S and the 18S ribosomal RNA fragments, respectively. L is the ladder column.
Supplementary 1. Electropherograms—generated by the Agilent Technologies’ 2100 Bioanalyzer system using an RNA 6000 Nano kit—from wild-type C. elegans exposed to different concentrations of BaP (0, 5, and 20 μM) for 48 h, showing four RNA biological replicate samples for each group (A, B, C, and D). All samples contained DMSO (0.1% v/v). The peak closest to the 40-second mark is representative of the 18S ribosomal RNA fragment, and the peak closest to the 45-second mark is representative of the 26S ribosomal RNA fragment of C. elegans. The RNA integrity number (RIN) is also shown; RIN values usually range from 10 (intact) to 1 (totally degraded).

## Slide 2
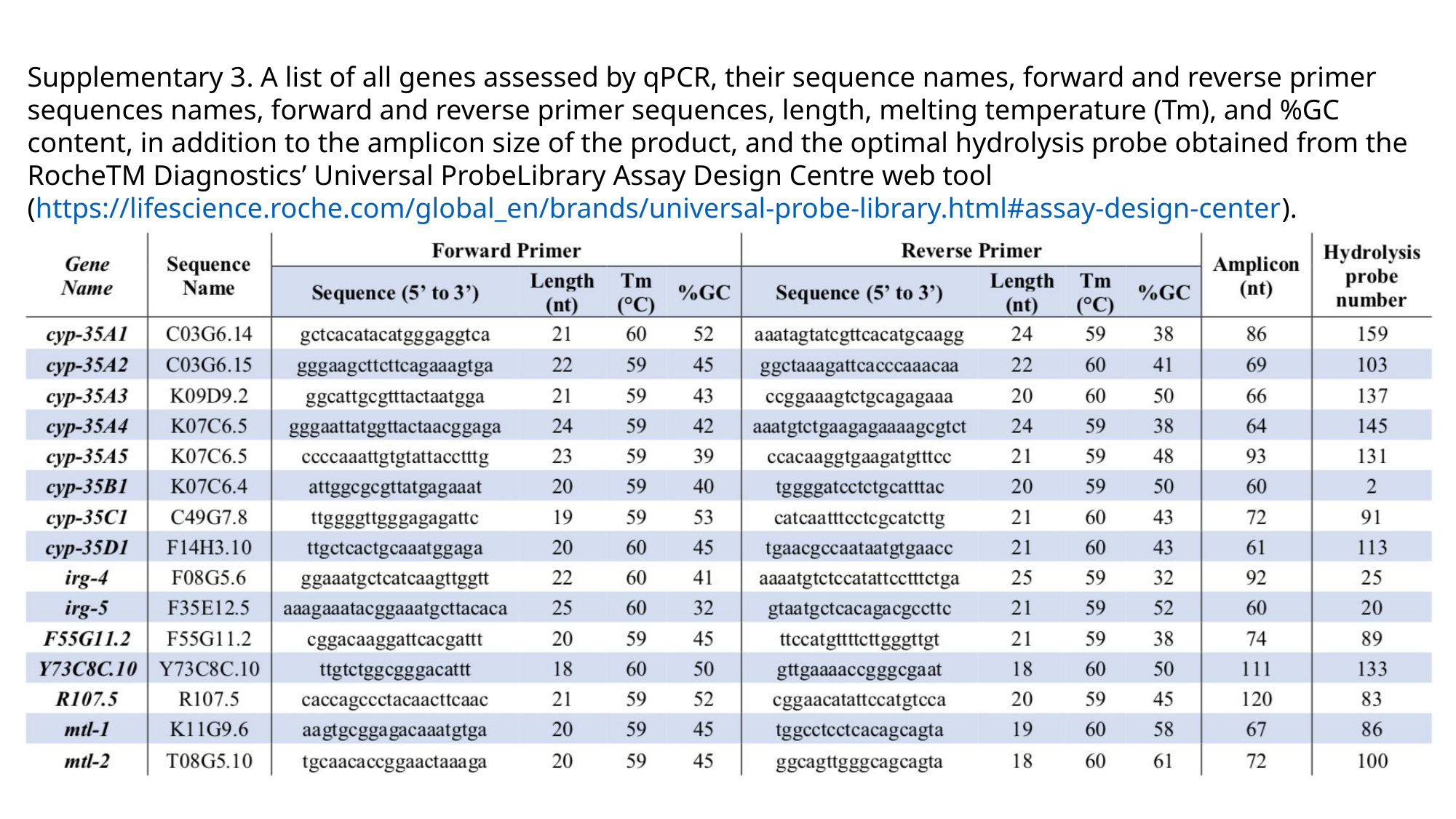

Supplementary 3. A list of all genes assessed by qPCR, their sequence names, forward and reverse primer sequences names, forward and reverse primer sequences, length, melting temperature (Tm), and %GC content, in addition to the amplicon size of the product, and the optimal hydrolysis probe obtained from the RocheTM Diagnostics’ Universal ProbeLibrary Assay Design Centre web tool (https://lifescience.roche.com/global_en/brands/universal-probe-library.html#assay-design-center).

## Slide 3
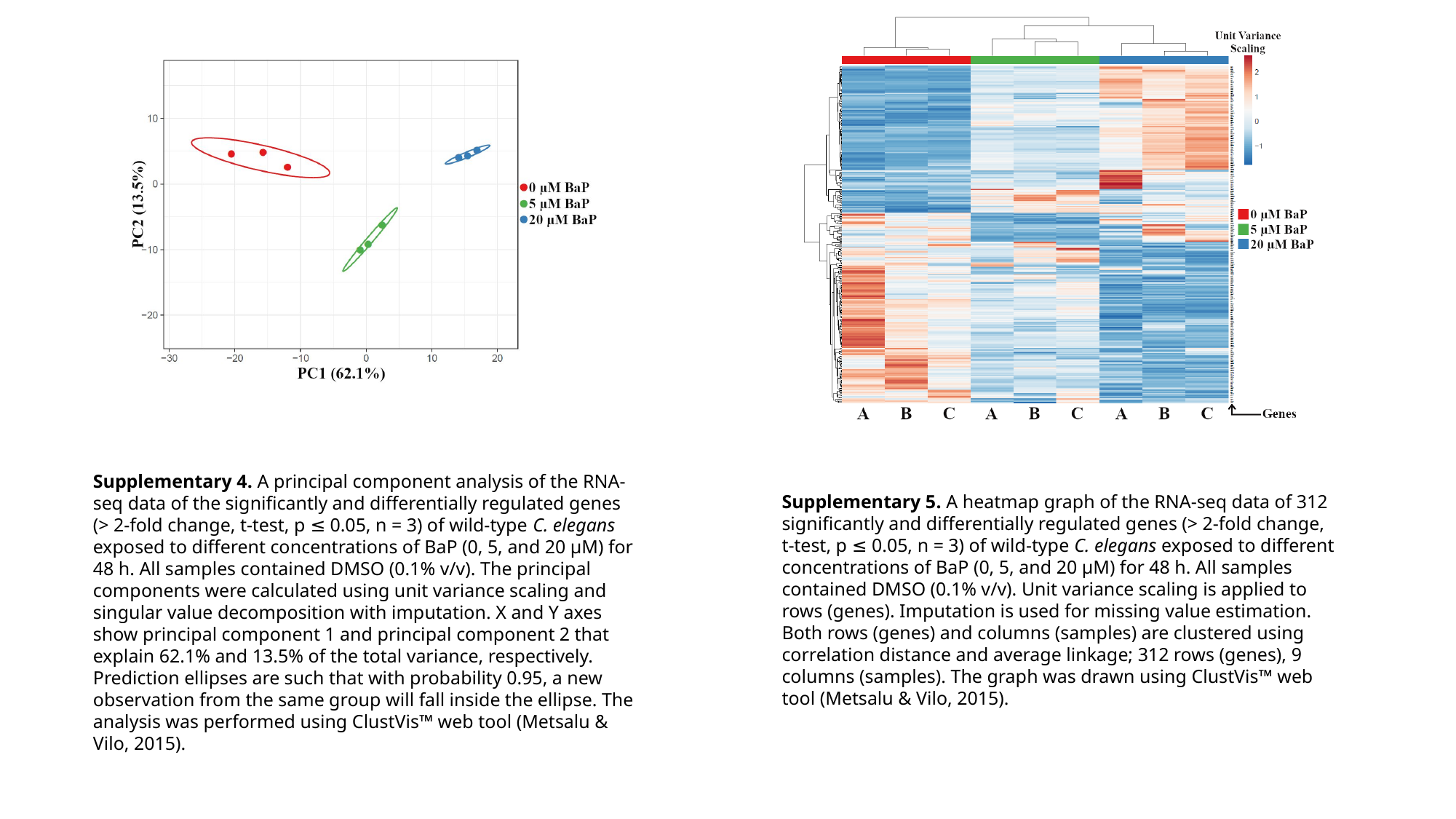

Supplementary 4. A principal component analysis of the RNA-seq data of the significantly and differentially regulated genes (> 2-fold change, t-test, p ≤ 0.05, n = 3) of wild-type C. elegans exposed to different concentrations of BaP (0, 5, and 20 µM) for 48 h. All samples contained DMSO (0.1% v/v). The principal components were calculated using unit variance scaling and singular value decomposition with imputation. X and Y axes show principal component 1 and principal component 2 that explain 62.1% and 13.5% of the total variance, respectively. Prediction ellipses are such that with probability 0.95, a new observation from the same group will fall inside the ellipse. The analysis was performed using ClustVis™ web tool (Metsalu & Vilo, 2015).
Supplementary 5. A heatmap graph of the RNA-seq data of 312 significantly and differentially regulated genes (> 2-fold change, t-test, p ≤ 0.05, n = 3) of wild-type C. elegans exposed to different concentrations of BaP (0, 5, and 20 µM) for 48 h. All samples contained DMSO (0.1% v/v). Unit variance scaling is applied to rows (genes). Imputation is used for missing value estimation. Both rows (genes) and columns (samples) are clustered using correlation distance and average linkage; 312 rows (genes), 9 columns (samples). The graph was drawn using ClustVis™ web tool (Metsalu & Vilo, 2015).

## Slide 4
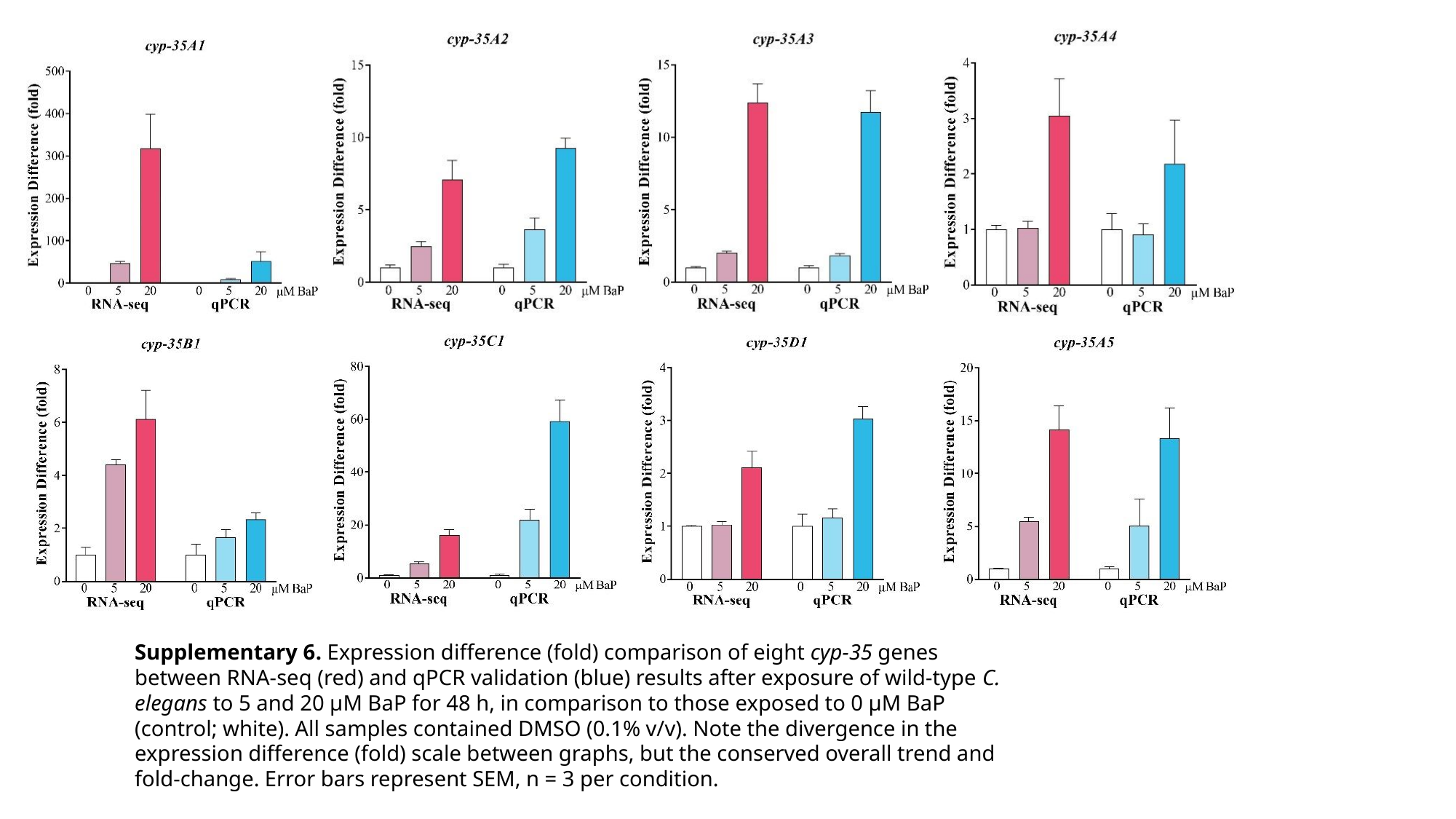

Supplementary 6. Expression difference (fold) comparison of eight cyp-35 genes between RNA-seq (red) and qPCR validation (blue) results after exposure of wild-type C. elegans to 5 and 20 µM BaP for 48 h, in comparison to those exposed to 0 µM BaP (control; white). All samples contained DMSO (0.1% v/v). Note the divergence in the expression difference (fold) scale between graphs, but the conserved overall trend and fold-change. Error bars represent SEM, n = 3 per condition.

## Slide 5
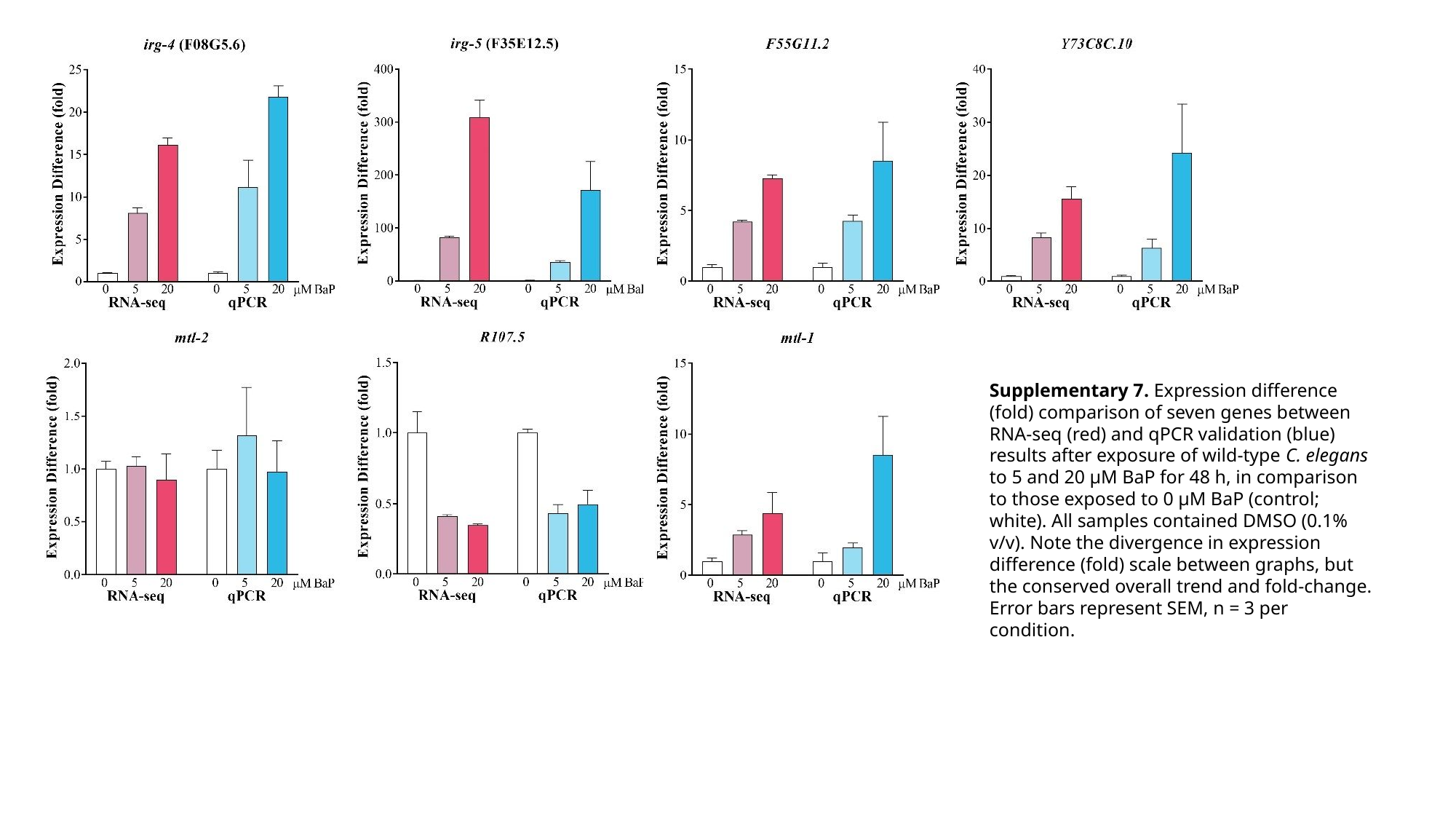

Supplementary 7. Expression difference (fold) comparison of seven genes between RNA-seq (red) and qPCR validation (blue) results after exposure of wild-type C. elegans to 5 and 20 µM BaP for 48 h, in comparison to those exposed to 0 µM BaP (control; white). All samples contained DMSO (0.1% v/v). Note the divergence in expression difference (fold) scale between graphs, but the conserved overall trend and fold-change. Error bars represent SEM, n = 3 per condition.

## Slide 6
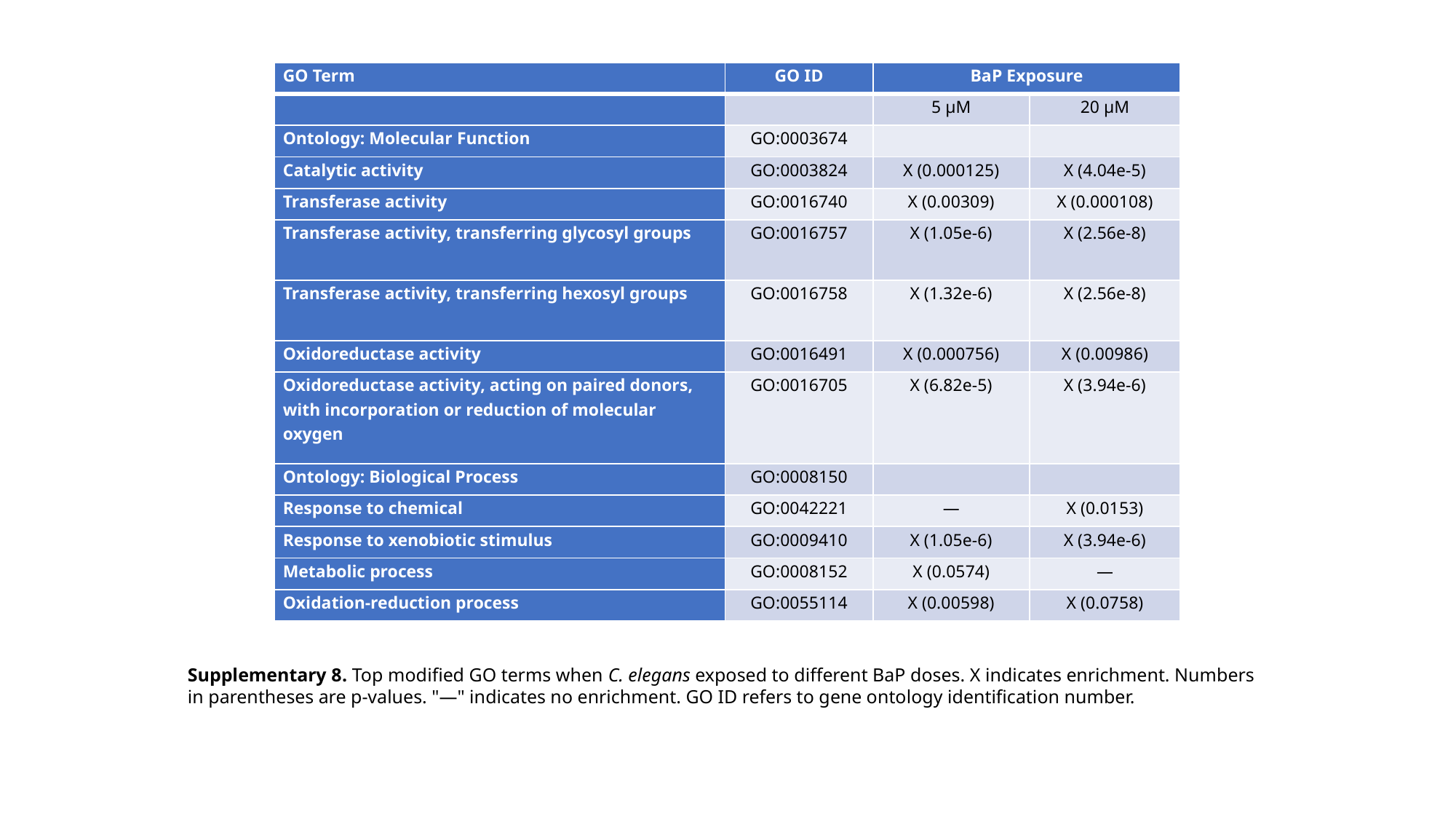

| GO Term | GO ID | BaP Exposure | |
| --- | --- | --- | --- |
| | | 5 µM | 20 µM |
| Ontology: Molecular Function | GO:0003674 | | |
| Catalytic activity | GO:0003824 | X (0.000125) | X (4.04e-5) |
| Transferase activity | GO:0016740 | X (0.00309) | X (0.000108) |
| Transferase activity, transferring glycosyl groups | GO:0016757 | X (1.05e-6) | X (2.56e-8) |
| Transferase activity, transferring hexosyl groups | GO:0016758 | X (1.32e-6) | X (2.56e-8) |
| Oxidoreductase activity | GO:0016491 | X (0.000756) | X (0.00986) |
| Oxidoreductase activity, acting on paired donors, with incorporation or reduction of molecular oxygen | GO:0016705 | X (6.82e-5) | X (3.94e-6) |
| Ontology: Biological Process | GO:0008150 | | |
| Response to chemical | GO:0042221 | — | X (0.0153) |
| Response to xenobiotic stimulus | GO:0009410 | X (1.05e-6) | X (3.94e-6) |
| Metabolic process | GO:0008152 | X (0.0574) | — |
| Oxidation-reduction process | GO:0055114 | X (0.00598) | X (0.0758) |
Supplementary 8. Top modified GO terms when C. elegans exposed to different BaP doses. X indicates enrichment. Numbers in parentheses are p-values. "—" indicates no enrichment. GO ID refers to gene ontology identification number.

## Slide 7
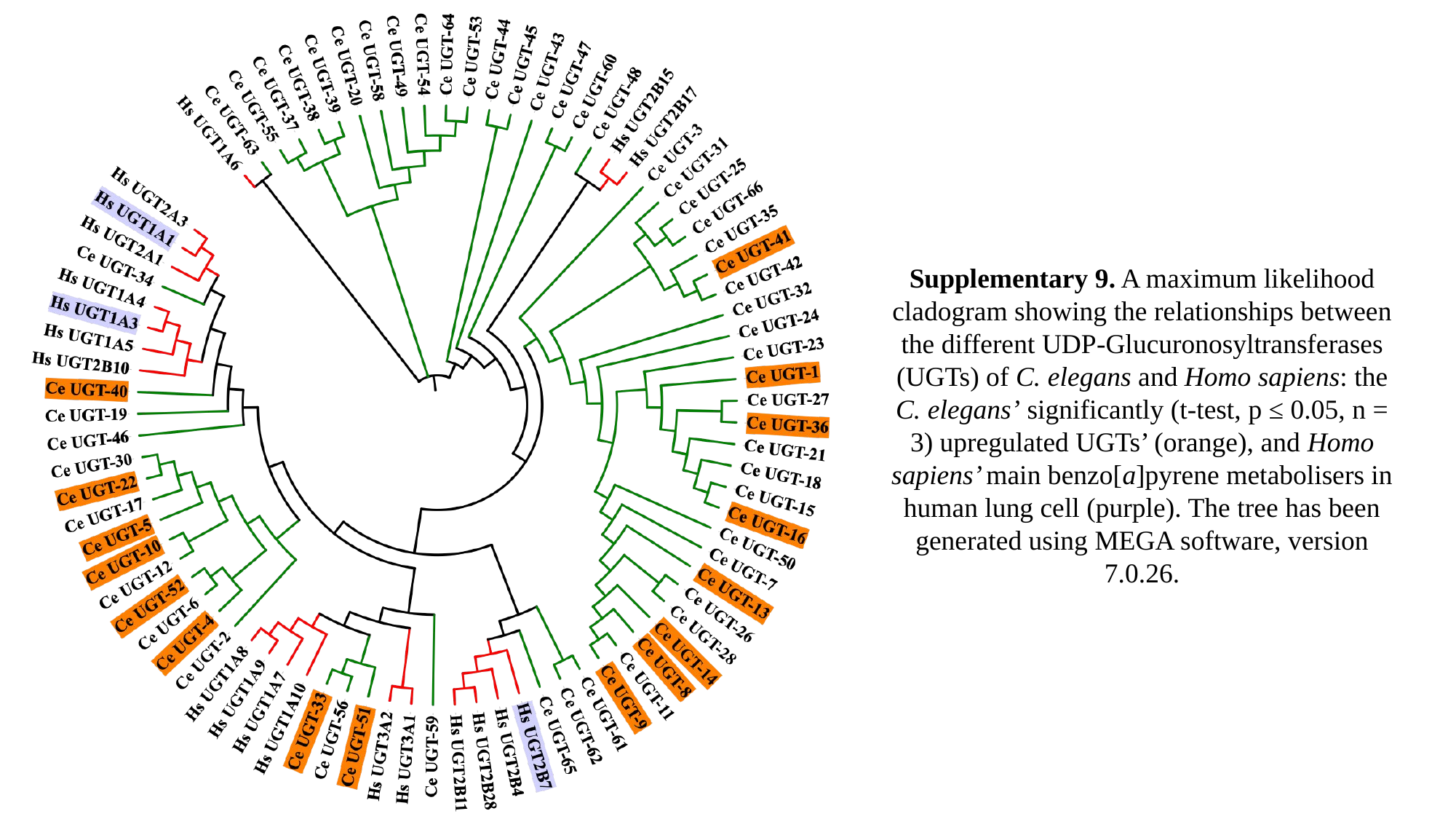

Supplementary 9. A maximum likelihood cladogram showing the relationships between the different UDP-Glucuronosyltransferases (UGTs) of C. elegans and Homo sapiens: the C. elegans’ significantly (t-test, p ≤ 0.05, n = 3) upregulated UGTs’ (orange), and Homo sapiens’ main benzo[a]pyrene metabolisers in human lung cell (purple). The tree has been generated using MEGA software, version 7.0.26.

## Slide 8
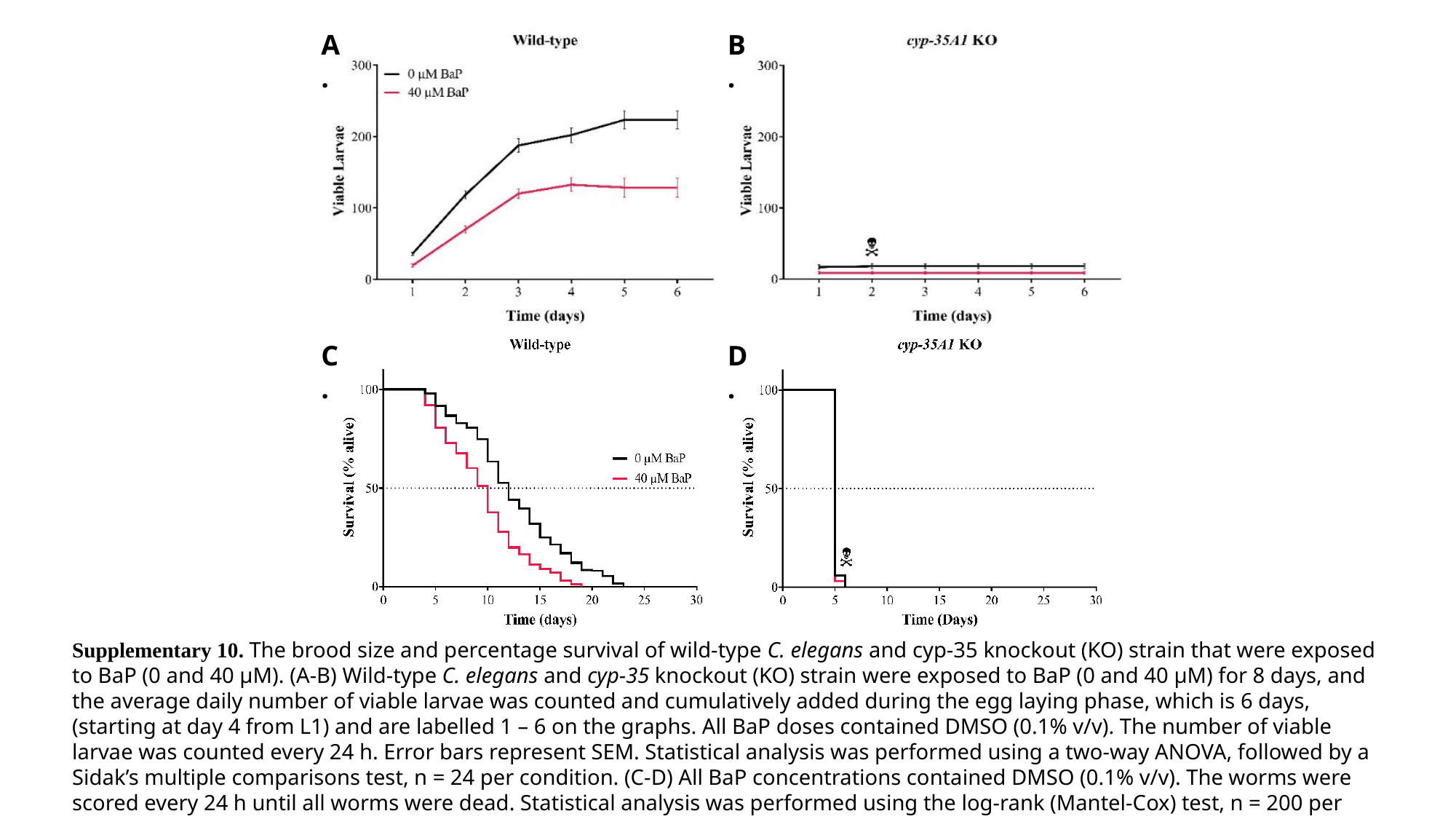

A.
B.
C.
D.
Supplementary 10. The brood size and percentage survival of wild-type C. elegans and cyp-35 knockout (KO) strain that were exposed to BaP (0 and 40 µM). (A-B) Wild-type C. elegans and cyp-35 knockout (KO) strain were exposed to BaP (0 and 40 µM) for 8 days, and the average daily number of viable larvae was counted and cumulatively added during the egg laying phase, which is 6 days, (starting at day 4 from L1) and are labelled 1 – 6 on the graphs. All BaP doses contained DMSO (0.1% v/v). The number of viable larvae was counted every 24 h. Error bars represent SEM. Statistical analysis was performed using a two-way ANOVA, followed by a Sidak’s multiple comparisons test, n = 24 per condition. (C-D) All BaP concentrations contained DMSO (0.1% v/v). The worms were scored every 24 h until all worms were dead. Statistical analysis was performed using the log-rank (Mantel-Cox) test, n = 200 per BaP concentration per strain.
